# Role of interleukin-23 in the development of nonallergic eosinophilic inflammation in a murine model of asthma

**DOI:** 10.1038/s12276-019-0361-9

**Published:** 2020-01-20

**Authors:** Hyun Seung Lee, Da-Eun Park, Ji-Won Lee, Kyung Hee Sohn, Sang-Heon Cho, Heung-Woo Park

**Affiliations:** 1 Institute of Allergy and Clinical Immunology, Seoul National University Medical Research Center, Seoul, Republic of Korea; 2 Division of Allergy and Clinical Immunology, Department of Asan Medical Center, University of Ulsan College of Medicine, Seoul, Republic of Korea; 3 Department of Internal Medicine, Kyung Hee University Medical Center, Seoul, Republic of Korea; 4 Department of Internal Medicine, Seoul National University College of Medicine, Seoul, Republic of Korea

**Keywords:** Asthma, Innate immunity

## Abstract

Nonallergic eosinophilic asthma (NAEA) is a clinically distinct subtype of asthma. Thus far, the pathophysiologic mechanisms underlying NAEA have not been fully elucidated. This study aimed to determine the role of IL-23 in the pathogenesis of NAEA. We developed a murine model of NAEA using recombinant IL-23 (rIL-23) plus a nonspecific airway irritant [polyinosinic-polycytidylic acid (polyI:C) or diesel exhaust particles (DEPs)] and investigated whether IL-23 plays an important role in the development of NAEA. Intranasal administration of rIL-23 (0.1 μg/mouse) plus polyI:C (0.01 μg/mouse) or DEPs (10 μg/mouse) without allergen resulted in methacholine bronchial hyperresponsiveness and eosinophilic airway inflammation in mice, which are characteristic features of NAEA. rIL-23 plus a low dose nonspecific airway irritants induced the release of innate cytokines from airway epithelium, including IL-33, thymic stromal lymphopoietin and IL-1β; these factors activated types 2 and 3 innate lymphoid cells (ILC2s and ILC3s). ILC2s and ILC3s, but not CD4+ T cells (i.e., adaptive immune cells), were important in the development of NAEA. In addition, we observed that IL-23 receptor expressions increased in airway epithelial cells, which suggests the existence of a positive autocrine loop in our murine model of NAEA. To our knowledge, this is the first report in which administration of rIL-23 plus a nonspecific airway irritant (polyI:C or DEPs) without allergen resulted in features of NAEA in mice similar to those found in humans. IL-23 may constitute a therapeutic target for NAEA in humans.

## Non-allergic asthma: Possible therapeutic target identified

Targeting levels of a pro-inflammatory protein may help quell responses to airway irritants in patients with non-allergic asthma. Asthma often occurs when allergen exposure triggers an increase in white blood cells called eosinophils and the subsequent release of pro-inflammatory proteins such as interleukin-23 (IL-23) in the airways. However, research suggests up to one-third of sufferers have non-allergic eosinophilic asthma (NAEA), wherein airway inflammation is triggered by no specific allergen. Heung-Woo Park at the Seoul National University Medical Research Center, South Korea, and co-workers created a mouse model with excess IL-23 to examine the protein’s role in NAEA inflammation. They monitored airway responses to low doses of an acid irritant or diesel exhaust particles. The combination of high IL-23 plus an irritant triggered the release of other pro-inflammatory proteins in the airways, aggravating asthma symptoms.

## Introduction

The classical paradigm of asthma pathogenesis involves an allergic response: allergen-specific T helper type 2 (Th2) cells produce cytokines that regulate the allergen-specific synthesis of immunoglobulin E (IgE) and the recruitment of eosinophils^1^. However, in practice, some patients with asthma have negative skin prick test results when exposed to a panel of common allergens or have no allergen-specific IgE. This nonallergic asthma occurs in 10–33% of patients with asthma, and has a later onset than allergic asthma^2^.

Eosinophils, leukocytes with granules that can be stained by the acidic red dye eosin, have long been considered a component of allergic responses^3,4^. However, eosinophils are also found in nonallergic disorders, such as nonallergic conjunctivitis^5^ and nonallergic rhinitis^6^. Recent advances in the immunologic understanding of asthma pathogenesis have revealed that interactions between innate and adaptive immunity produce heterogenetic features, including nonallergic eosinophilic asthma (NAEA), a distinct subtype of asthma^7^.

However, little is known about the mechanism of the occurrence of NAEA. According to a recent study, innate immune cells, especially, innate lymphoid cells (ILCs), may play a more critical role in the development of NAEA than adaptive immune cells^8^.

For example, in a murine model without allergen administration, influenza virus infection induced airway hyperresponsiveness (AHR) through ILC2s via the interleukin (IL)-33/IL-13 axis, independent of Th2 cells^9^. In addition, activated ILC2s can cause eosinophilia by producing interleukin IL-5^7,10^. As reported before, nonspecific irritants, such as air pollutants and microbes, are able to induce ILC2s^11^. However, thus far, there has been no murine model of asthma showing both AHR and eosinophilic airway inflammation without allergen administration.

IL-23 is a proinflammatory cytokine that is known to play an important role in the development of numerous autoimmune diseases by integrating the innate and adaptive immune systems^12^. Notably, excess production of IL-23 or transgenic overexpression of the IL-23 receptor has been shown to exacerbate allergen-induced eosinophilic inflammation through Th2 (CD4) cell-dependent mechanisms^13^. We and other colleagues showed an essential role of IL-23 that is released from airway epithelium in the development of ILC2-mediated eosinophilic inflammation using a house dust mite-induced murine model of asthma^14,15^. However, the role of IL-23 in NAEA is unknown.

Given that IL-23 secretion is enhanced in response to nonspecific airway irritants, such as lipopolysaccharide, viral infection, and urban particulate matter^16–18^, we hypothesized that IL-23 plays an important role in the development of NAEA. To test this hypothesis, we evaluated phenotypic differences among mice that were intranasally administered recombinant IL-23 (rIL-23) plus low dose polyinosinic-polycytidylic acid (poly I:C, a mimic of viral infection), rIL-23 plus diesel exhaust particles (DEPs), or phosphate-buffered saline (PBS) control treatment.

## Materials and methods

### rIL-23 stimulation experiments

**Fig. 1:**
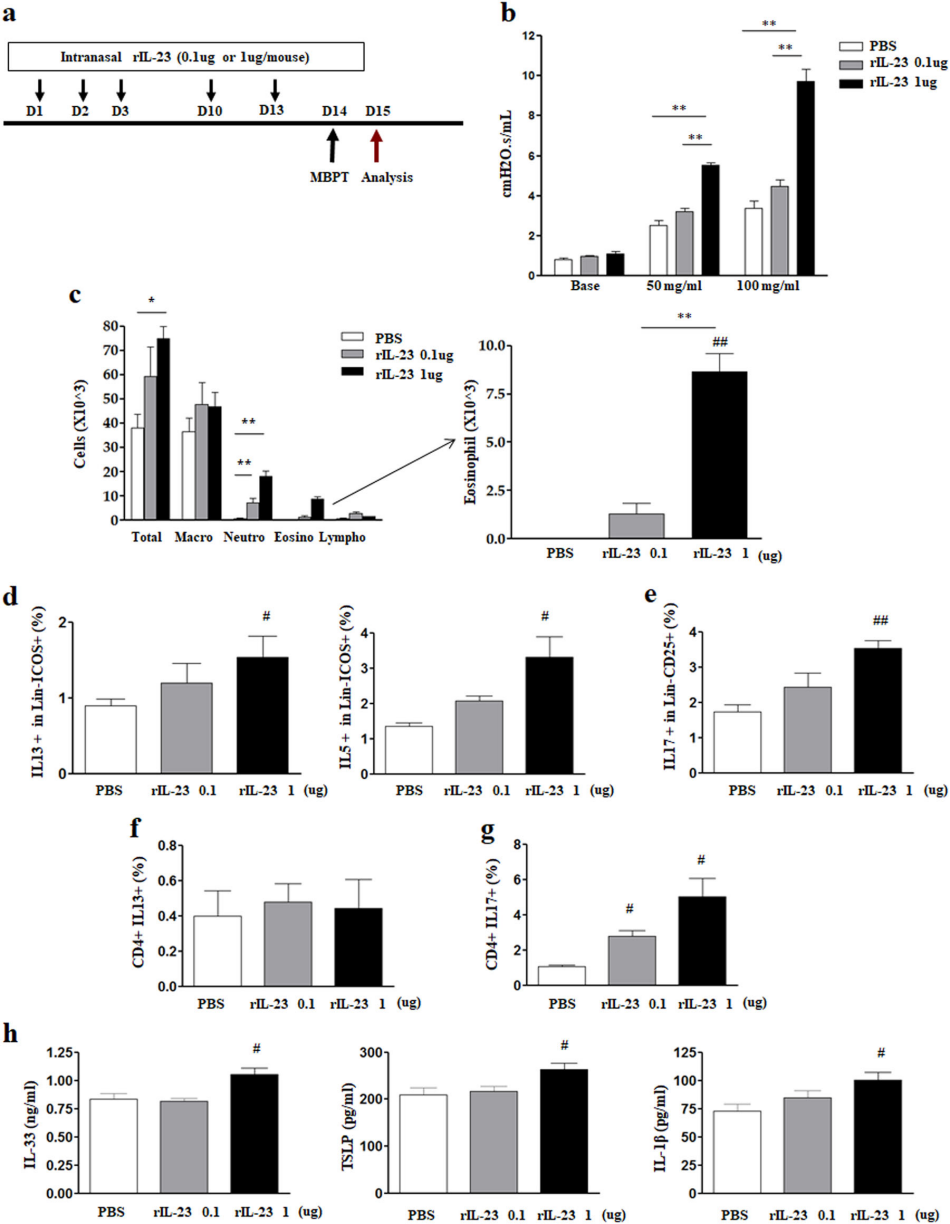
Determination of recombinant interleukin-23 (rIL-23) dose for stimulation. **a** Experimental protocol. **b** Airway resistance at doses of 50 and 100 mg/mL methacholine (MCh). **c** Inflammatory cells in bronchoalveolar lavage fluid (eosinophils are magnified). **d** IL-13-producing or IL-5-producing type 2 innate lymphoid cells (CD45+Lin− ICOS+) in lung homogenate. **e** IL-17A-producing type 3 innate lymphoid cells (CD45+Lin− CD25+) in lung homogenate. **f**, **g** IL-13-producing or IL-17A-producing CD4+ T cells in lung homogenate. **h** IL-33 and TSLP levels in lung homogenate. *P < 0.05, **P < 0.01 between two groups; ^#^*P* < 0.05, ^##^*P* < 0.01 compared to the control group; AHR airway hyperresponsiveness; PBS phosphate-buffered saline; Macro macrophage; Neutro neutrophil; Eosino eosinophil; Lympho lymphocyte.

Six-week-old female BALB/C mice (18–20 g) were purchased from Orient Bio (Seoul, Korea). All experiments were performed with the approval of the Institutional Animal Care and Use Committee of the Institute of Laboratory Animal Resources at Seoul National University (SNU-170123-4-2). We evaluated phenotypic changes in mice induced by intranasal administration of various doses of rIL-23 (eBioscience, San Diego, CA, USA) without allergens (0.1 or 1 μg/mouse, five times over a period of 2 weeks), based on a previous report (Fig. 1a)^19^. One day after the final administration, methacholine AHR was measured using invasive methods (details are presented in the Supplementary information). The mice were then sacrificed to analyze bronchoalveolar lavage (BAL) fluid and lung tissue. We selected 0.1 μg/mouse as the rIL-23 dose in subsequent experiments, as this dose did cause significant changes in airway inflammation or immune responses.

### Murine model of NAEA

Two experimental models were generated: polyI:C/rIL-23 (0.01 μg/mouse of polyI:C [Sigma-Aldrich, St. Louis, MO, USA] plus 0.1 μg/mouse of rIL-23) and DEP/rIL-23 (10 μg/mouse of DEPs [SRM 2975, NIST, Gaithersburg, MD, USA] plus 0.1 μg/mouse of rIL-23). Based on previous reports, we selected doses of polyI:C and DEPs that would not cause airway inflammation by themselves^20,21^. DEPs were suspended in PBS with 0.5% dimethyl sulfoxide. The mice were divided into six treatment groups: PBS (control), polyI:C, DEP, rIL-23, polyI:C/rIL-23, and DEP/rIL-23. Treatments were performed on days 1, 2, 3, 14, 15, 21, and 22; the mice were then sacrificed for evaluation of methacholine AHR, inflammatory cells in BAL fluid, and histology (Fig. S1).

### Measurements of ILCs, Th cells, and innate cytokines

Single cells prepared from lung tissue were stimulated with phorbol 12-myristate 13-acetate (100 ng/mL), ionomycin (1 μg/mL), and Golgi stop, and then stained with PerCP-Cy5.5-conjugated anti-cluster of differentiation (CD) 45 antibodies (eBioscience). To isolate ILCs, flow cytometry of lineage-negative cells was performed with gating for fluorescein isothiocyanate (FITC)-conjugated antibodies against CD3, CD4, CD8, CD11b, CD11c, CD19, F4/80, FcεRI, and CD49b. Allophycocyanin (APC)-conjugated anti-inducible T cell costimulator (ICOS) antibodies (eBioscience) and APC-conjugated anti-CD25 antibody (eBioscience) were also used for analysis. For intracellular staining, cells were permeabilized (Cytofix/Cytoperm kit; BD Biosciences, San Jose, CA, USA) and incubated with phycoerythrin (PE)-Cy7-conjugated anti-IL-13 antibody (eBioscience), PE-conjugated anti-IL-5 antibodies (BD Biosciences), or cyan fluorescent protein (CFP)-conjugated anti-IL-17A antibodies (eBioscience). To quantify Th2 or Th17 cells, the cells were stained with PerCP-Cy5-conjugated anti-CD4 antibodies and PE-Cy7-conjugated anti-IL-13 or CFP-conjugated anti-IL-17A antibodies. For flow cytometry analysis, at least 3 × 10^5^ cells were acquired using an LSR II (BD Biosciences). In the supernatants of crushed and homogenized mouse lungs, levels of IL-23 (BioLegend, San Diego, CA, USA), IL-1β (BioLegend), thymic stromal lymphopoietin (TSLP; BioLegend), and IL-33 (R&D Systems, Abingdon, UK) were measured using enzyme-linked immunosorbent assay (ELISA), in accordance with the manufacturer’s instructions. Our gating strategy for flow cytometry analysis is presented in Fig. S2.

### Measurements of eosinophils in BAL fluid and IL-23 receptor expression in lung cells

To quantify eosinophils in BAL fluid, cells were stained with FITC-conjugated anti-CD11b antibodies (eBioscience), APC/Cy7-conjugated anti-CD11c antibodies (eBioscience), and PE-conjugated anti-Siglec-F antibodies (BD Biosciences). To measure IL-23 receptor (IL-23R) expression in lung cells, single cells prepared from lung tissue were stained with PerCP-Cy5.5-conjugated anti-CD45 antibodies, FITC-conjugated anti-F4/80 antibodies (eBioscience), APC/Cy7-conjugated anti-CD11c antibodies, APC-conjugated anti-epithelial cell adhesion molecule (EpCAM) antibodies (eBioscience), and PE-conjugated anti-IL-23R antibodies (eBioscience).

### Immunohistochemistry (IHC) staining

Lung sections were incubated with the following primary antibodies: anti-α-smooth muscle actin (SMA, 1:200; Abcam, Cambridge, UK), anti-IL-33 (1:50; R&D Systems, Minneapolis, MN, USA), anti-TSLP (1:4,000; Abcam), and anti-IL-23R (1:200; Abcam). For isotype controls, anti-rabbit or anti-goat IgG antibodies were used. IHC staining was photographed using a Nikon light microscope and analyzed with digital imaging software (iSolution Lite, IMT i-Solution Inc., Daejeon, Korea). Slides were examined at ×400 magnification. The average percent area of IHC stainings was quantified as positive areas with 4–5 fields/mouse (*n* = 3) using ImageJ software (NIH, Bethesda, MD, USA) after setting the thresholds.

### Effects of polyI:C, DEP, and rIL-23 treatments on MLE12 cells

For further insight, the effects of polyI:C, DEP, or rIL-23 treatment were assessed using a mouse lung epithelial cell line. MLE12 cells (SV40-transformed mouse-derived alveolar epithelial cell line; American Type Culture Collection, Manassas, VA, USA) were grown in Dulbecco’s modified Eagle medium:Ham’s F-12 with 2% fetal bovine serum in a humidified atmosphere at 37 °C with 5% CO_2_; the cells were then stimulated with different doses of polyI:C (0.01 or 50 μg/mL) or different doses of DEPs (0.01 or 0.1 μg/mL), with or without anti-IL-23p19 antibodies (0.05 μg/mL), for 24 h. The effects of different doses of rIL-23 (0.002 and 0.01 μg/mL) on MLE12 cells were also evaluated. Levels of cytosolic IL-23, nuclear IL-33, and cytosolic TSLP were determined using ELISA (Nuclear/Cytosol Fractionation Kit [NE-PER], Pierce Biotechnology, Rockford, IL, USA).

### Coculture of ILC2s with polyI:C/rIL-23-treated or DEP/rIL-23-treated MLE12 cells

The effects of exposure to polyI:C/rIL-23-treated or DEP/rIL-23-treated MLE12 cells on ILC2s were assessed using indirect coculture. Direct interactions were prevented by using Transwell inserts (pore size 0.4 μM; BD Biosciences), which separated the cells into two compartments. MLE12 cells were seeded in the upper chambers of a 12-well plate (2 × 10^5^/well), treated with polyI:C (0.01 μg/mL) or DEP (0.01 μg/mL) with or without rIL-23 (0.002 μg/mL) for 24 h, and washed with PBS. ILC2s were collected from mice treated with recombinant IL-33 (0.5 μg/mouse, eBioscience) for 5 days (Fig. S3). ILC2s were placed in the lower chambers of the plates and incubated for 48 h with MLE12 cells treated as detailed above. Culture supernatants were collected and IL-5 and IL-13 levels were determined by using ELISA. Then, to investigate the importance of IL-23R signaling, we performed an IL-23R transfection experiment. MLE12 cells (1 × 10^5^/well) were maintained overnight in Dulbecco’s modified Eagle medium: Ham’s F-12 with 2% fetal bovine serum and then transfected with 0.2 μg plasmid IL-23R (IL-23R Gene ORF cDNA clone expression plasmid; Sino Biological, Beijing, China) with Sinofection reagent (Sino Biological), in accordance with the manufacturer’s instructions. After 2 days of culture, IL-23R transfection was confirmed using a western blot assay (Fig. S4).

### Statistics

The results are expressed as the means ± standard deviation, and significant differences among groups were assessed using the Kruskal–Wallis test or the Mann–Whitney *U* test. For multiple comparisons, the Kruskal–Wallis test was used initially; if significant differences were found, the Mann–Whitney *U* test was then used to determine differences between pairs of groups. There were six mice in each group, and triplicate results are presented. Statistical analyses were performed using GraphPad Prism 4.01 (GraphPad Software, La Jolla, CA, USA). *P*-values < 0.05 were considered significant.

## Results

### rIL-23 stimulation experiments

Methacholine AHR significantly increased only in mice treated with 1 μg/mouse rIL-23 (Fig. 1b). The number of neutrophils and eosinophils in the BAL fluid increased in a dose-dependent manner in rIL-23-treated mice (Fig. 1c). However, a significant increase in the number of eosinophils was observed only in mice treated with 1 μg/mouse rIL-23. In lung cells, the number of IL-13 or IL-5-producing ILC2s (CD45+Lin−ICOS+) and IL-17-producing ILC3s (CD45+Lin−CD25+) significantly increased in mice treated with 1 μg/mouse rIL-23 (Fig. 1d, e, S5a–c). The numbers of IL-13-producing CD4+ T cells in lung homogenate did not significantly differ among treatment groups, whereas the numbers of IL-17-producing CD4+ T cells significantly increased in a dose-dependent manner in rIL-23-treated mice (Fig. 1f, g, S5d, e). The levels of IL-33, TSLP, and IL-1β in lung homogenate were significantly higher in mice treated with 1 μg/mouse rIL-23 than in control mice (Fig. 1h). Based on these observations, we selected 0.1 μg/mouse for rIL-23 dose in subsequent experiments.

### polyI:C/rIL-23 model

**Fig. 2:**
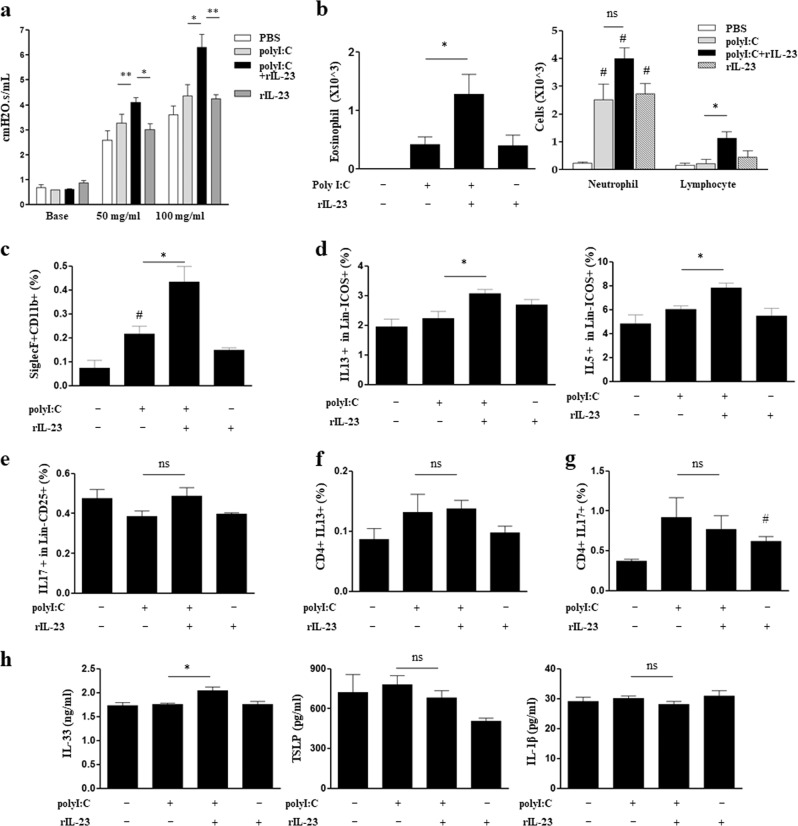
Nonallergic eosinophilic asthma phenotype of the polyI:C/rIL-23 model. **a** Airway resistance at doses of 50 and 100 mg/mL methacholine (MCh). **b** Eosinophils, neutrophils, and lymphocytes in bronchoalveolar lavage fluid. **c** CD11b+siglecF+CD11c− cells in bronchoalveolar lavage fluid. **d** IL-13-producing or IL-5-producing type 2 innate lymphoid cells (CD45+Lin− ICOS+) in lung homogenate. **e** IL-17A-producing type 3 innate lymphoid cells (CD45+Lin− CD25+) in lung homogenate. **f**, **g** IL-13-producing or IL-17A-producing CD4+ T cells in lung homogenate. **h** IL-33, TSLP, and IL-1β levels in lung homogenate. *P < 0.05, **P < 0.01 between two groups; ^#^*P* < 0.05 compared to the control group; ns, not significant; PBS phosphate-buffered saline.

Methacholine AHR and the number of eosinophils in the BAL fluid were significantly higher in the polyI:C/rIL-23 group than in the polyI:C and rIL-23 groups (Fig. 2a, b). The number of neutrophils increased significantly in the BAL fluid of all three treatment groups, compared to that of the PBS group; however, the number of neutrophils did not significantly differ between the polyI:C and polyI:C/rIL-23 groups (Fig. 2b). An increased number of eosinophils (CD11b+siglecF+CD11c−) was confirmed again using flow cytometry (Fig. 2c, S6a). The populations of lymphocytes associated with innate or adaptive immunity in the BAL fluid were measured using flow-cytometry. The numbers of IL-13 or IL-5-producing ILC2s were significantly higher in the polyI:C/rIL-23 group than in the polyI:C group, whereas the numbers of IL-17-producing ILC3s did not differ between the two groups (Fig. 2d, e, S6b–d). The number of IL-13-producing CD4+ T cells did not differ among any of the groups; in contrast, IL-17-producing CD4+ T cells were significantly higher in the rIL-23 group than in the PBS group, whereas there was no difference in the number of IL-17-producing CD4+ T cells between the polyI:C/rIL-23 and polyI:C groups (Fig. 2f, g, S6e). The level of IL-33 in lung homogenate was significantly higher in the polyI:C/rIL-23 group than polyI:C group, but TSLP and IL-1β levels did not differ between the two groups (Fig. 2h).

### DEP/rIL-23 model

**Fig. 3:**
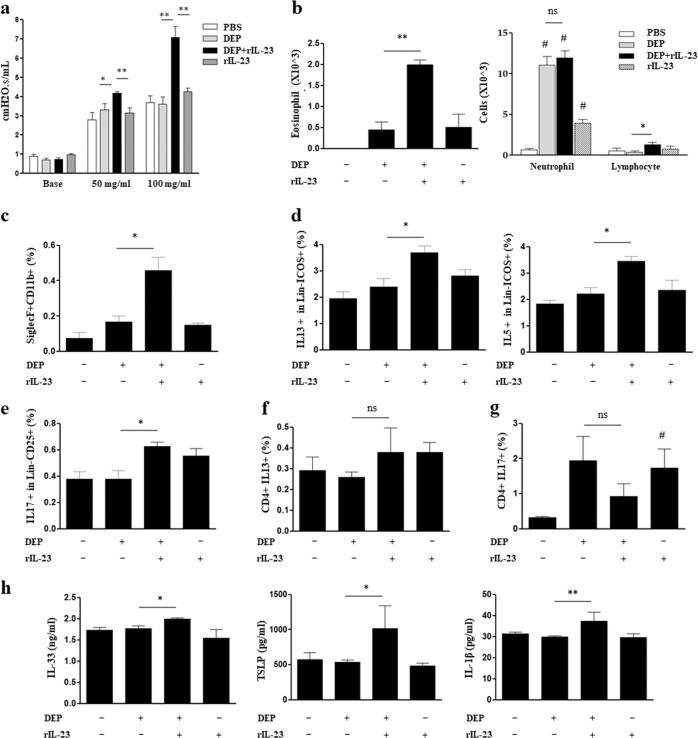
Nonallergic eosinophilic asthma phenotype of the DEP/rIL-23 model. **a** Airway resistance at doses of 50 and 100 mg/mL methacholine (MCh). **b** Eosinophils, neutrophils, and lymphocytes in bronchoalveolar lavage fluid. **c** CD11b+siglecF+CD11c− cells in bronchoalveolar lavage fluid. **d** IL-13 or IL5-producing type 2 innate lymphoid cells (CD45+Lin− ICOS+) in lung homogenate. **e** IL-17A-producing type 3 innate lymphoid cells (CD45+Lin− CD25+) in lung homogenate. **f**, **g** IL-13-producing or IL-17A-producing CD4+ T cells in lung homogenate. **h** IL-33, TSLP, and IL-1β levels in lung homogenate. *P *<* 0.05, **P *<* 0.01 between two groups; ^#^*P* *<* 0.05 compared to the control group; DEP diesel exhaust particle; ns, not significant; PBS phosphate-buffered saline.

Methacholine AHR and the number of eosinophils in the BAL fluid were significantly higher in DEP/rIL-23 group than in the DEP group (Fig. 3a, b). The numbers of neutrophils increased significantly in the BAL fluid of all three treatment groups, compared to that of the PBS group; however, the number of neutrophils did not significantly differ between the DEP and DEP/rIL-23 groups (Fig. 3b). An increased number of eosinophils (CD11b+siglecF+CD11c−) was confirmed again using flow cytometry (Fig. 3c, S7a). The number of IL-13-producing or IL-5-producing ILC2s and IL-17-producing ILC3s was significantly higher in the DEP/rIL-23 group than in the DEP group (Fig. 3d, e, S7b–d). Similar to the polyI:C/rIL-23 model, the number of IL-13-producing CD4+ T cells did not differ among any of the groups; moreover, the number of IL-17-producing CD4+ T cells was significantly higher in the rIL-23 group than in the PBS group, whereas there was no difference in the number of IL-17-producing CD4+ T cells between the DEP/rIL-23 and DEP groups (Fig. 3f, g, S7e). The levels of IL-33, TSLP, and IL-1β in lung homogenate were significantly higher in the DEP/rIL-23 group than in the DEP group (Fig. 3h).

### IHC staining

**Fig. 4:**
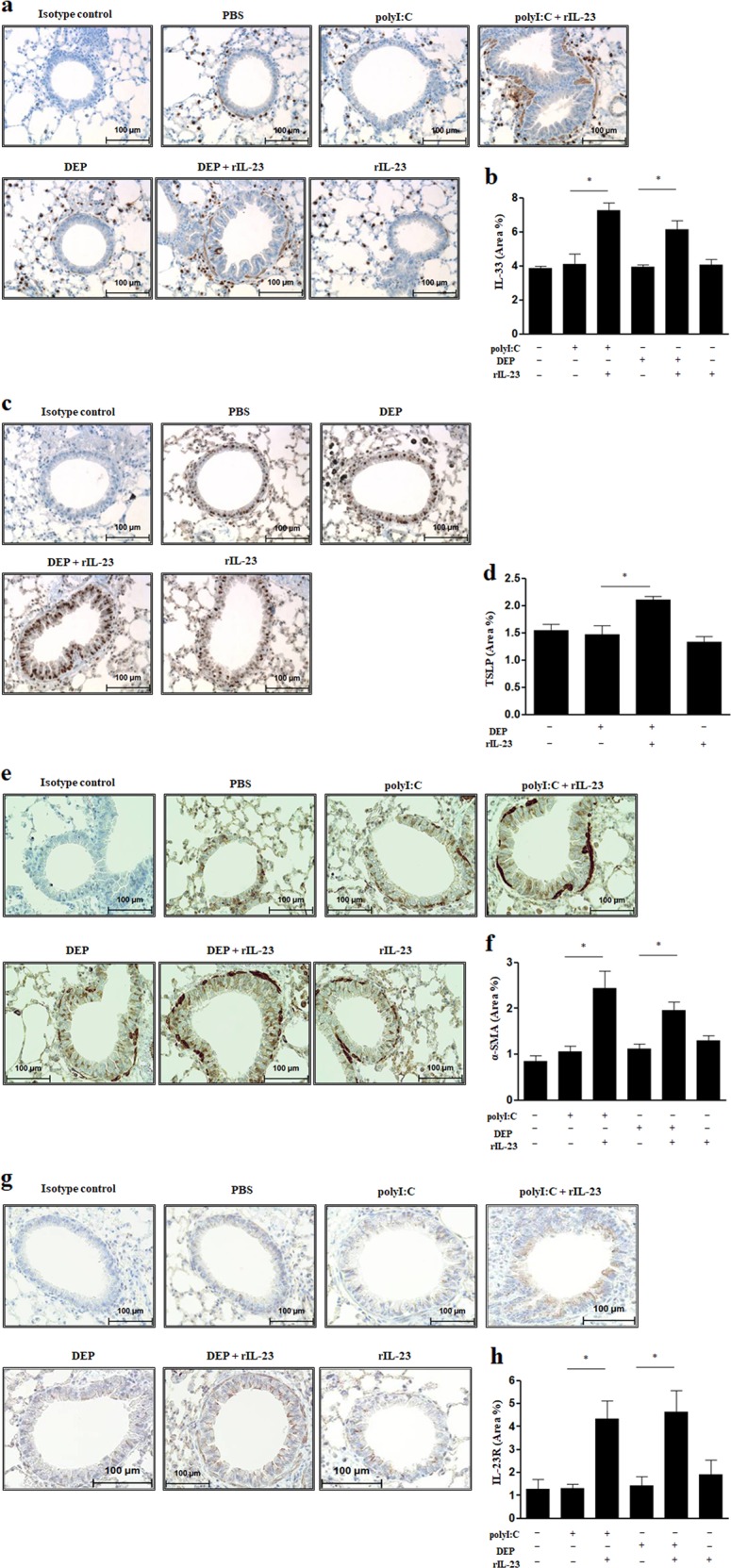
Immunohistochemistry findings. **a** IL-33. **b** Comparison of area% of IL-33. **c** TSLP. **d** Comparison of area% of TSLP. **e** α-SMA. **f** Comparison of area% of α-SMA. **g** IL-23R. **h** Comparison of area% of IL-23R. Magnification, ×40. Area% was quantified using ImageJ software. *P < 0.05 between two groups. DEP diesel exhaust particle; α-SMA alpha-smooth muscle actin; PBS phosphate-buffered saline.

The expression levels of IL-33 in the basal cellular layer of the epithelium were significantly higher in both the polyI:C/rIL-23 and DEP/rIL-23 groups than in the polyI:C, DEP, and rIL-23 groups (Fig. 4a, b). Moreover, TSLP expression levels significantly increased only in the DEP/rIL-23 group (Fig. 4c, d). The polyI:C/rIL-23 and DEP/rIL-23 groups showed profound enhancement of α-SMA expression compared to that of the other groups (Fig. 4e, f). IHC analysis of mouse lung tissue sections showed that IL-23R expression was higher in both the polyI:C/rIL-23 and DEP/rIL-23 groups than in the other groups (Fig. 4g, h). Using flow cytometry, we again confirmed that significantly increased IL-23R expression was present in mouse lung epithelial cells (EpCAM+CD45−), but not in CD11+F4/80-CD45+ cells or CD4+CD45+ cells (Fig. S8).

### Effect of polyI:C, DEP, and rIL-23 treatments on MLE12 cells

**Fig. 5:**
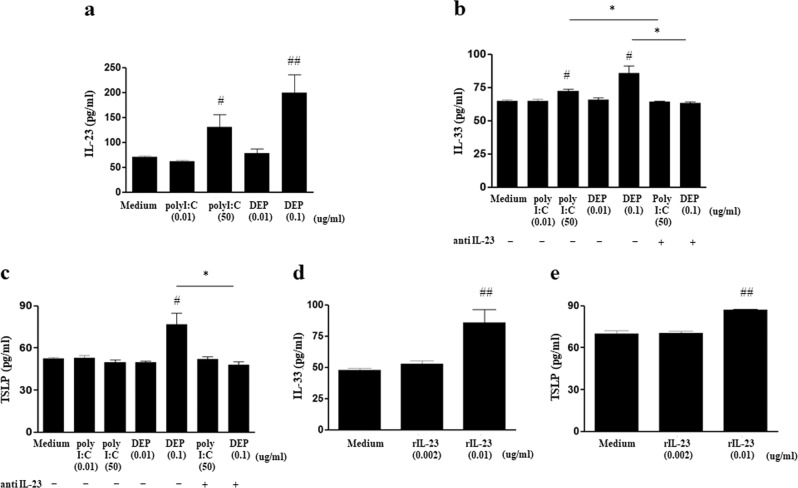
Effect of polyI:C, DEP, and rIL-23 treatments on MLE12 cells. **a** IL-23 production by MLE12 cells treated with different doses of polyI:C or DEP. **b** IL-33 production by MLE12 cells treated with different doses of polyI:C or DEPs, with or without anti-IL-23 antibodiesy. **c** TSLP production by MLE12 cells treated with different doses of polyI:C or DEPs, with or without anti-IL-23 antibodies. **d** IL-33 production by MLE12 cells treated with different doses of rIL-23. **e** TSLP production by MLE12 cells treated with different doses of rIL-23. *P < 0.05 between two groups; ^#^*P* < 0.05, ^##^*P* < 0.01 compared to medium only; DEP diesel exhaust particle.

MLE12 cells that were treated with 50 μg/mL polyI:C or 0.1 μg/mL DEPs showed significant increases in IL-23 production (Fig. 5a). IL-33 production significantly increased upon treatment with 50 μg/mL polyI:C or 0.1 μg/mL DEPs, but these increases were significantly attenuated with the addition of anti-IL-23p19 antibodies (Fig. 5b). Furthermore, a significant increase in TSLP production was observed only upon treatment with 0.1 μg/mL DEP, and was significantly attenuated with the addition of anti-IL-23p19 antibodies (Fig. 5c). Treatment with 0.01 μg/mL rIL-23 induced significant increases in the levels of IL-33 and TSLP produced by MLE12 cells (Fig. 5d, e).

### Effect of exposure to polyI:C/rIL-23-treated or DEP/rIL-23-treated MLE12 cells on ILCs

**Fig. 6:**
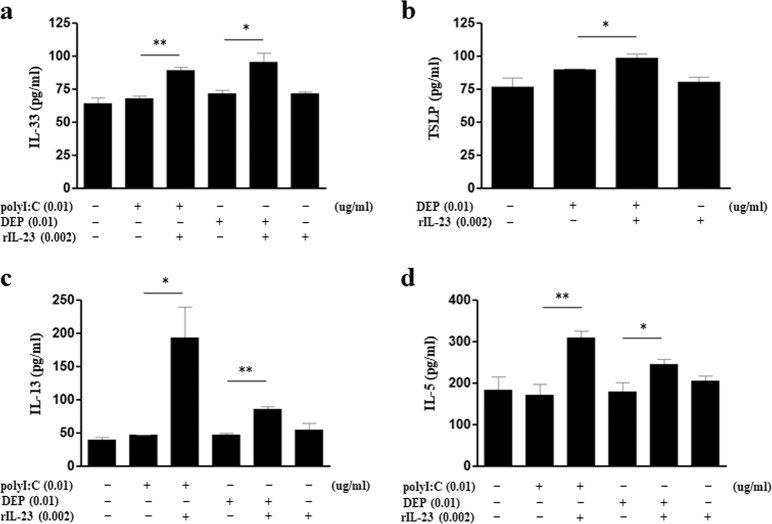
Effect of exposure to polyI:C/rIL-23-treated or DEP/rIL-23-treated MLE12 cells on ILCs. **a** IL-33 levels in the nuclei of MLE12 cells. **b** TSLP levels in the cytosol of MLE12 cells. **c** IL-13 levels in the supernatant of ILC2s cocultured with MLE12 cells treated with polyI:C or DEPs, with or without rIL-23. **d** IL-5 levels in the supernatant of ILC2s cocultured with MLE12 cells treated with polyI:C or DEPs, with or without rIL-23. *P < 0.05, **P < 0.01 between two groups; DEP diesel exhaust particle.

**Fig. 7:**
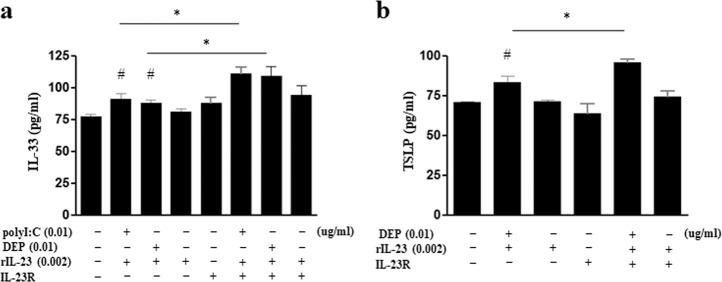
Effects of IL-23R overexpression in MLE12 cells treated with polyI:C/rIL-23 or DEP/rIL-23. **a** IL-33 levels in the nuclei of MLE12 cells. **b** TSLP levels in the cytosol of MLE12 cells. *P < 0.05 between two groups; ^#^*P* *<* 0.05 compared to medium only; DEP diesel exhaust particle.

IL-33 expression levels increased in both polyI:C/rIL-23-treated and DEP/rIL-23-treated MLE12 cells, whereas TSLP expression only increased in DEP/rIL-23-treated MLE12 cells (Fig. 6a, b). IL-5 and IL-13 levels in the supernatant of ILC2s cocultured with either polyI:C/rIL-23-treated MLE12 cells or DEP/rIL-23-treated MLE12 cells were significantly higher than those of ILC2s cocultured with either polyI:C-treated MLE12 cells (without rIL-23) or DEP-treated MLE12 cells (without rIL-23) (Fig. 6c, d). The levels of IL-33 and TSLP in polyI:C/rIL-23-treated or DEP/rIL-23-treated MLE12 cells overexpressing IL-23R were significantly higher than those of polyI:C/rIL-23-treated or DEP/rIL-23-treated MLE12 cells with normal IL-23R expression (Fig. 7).

## Discussion

**Fig. 8:**
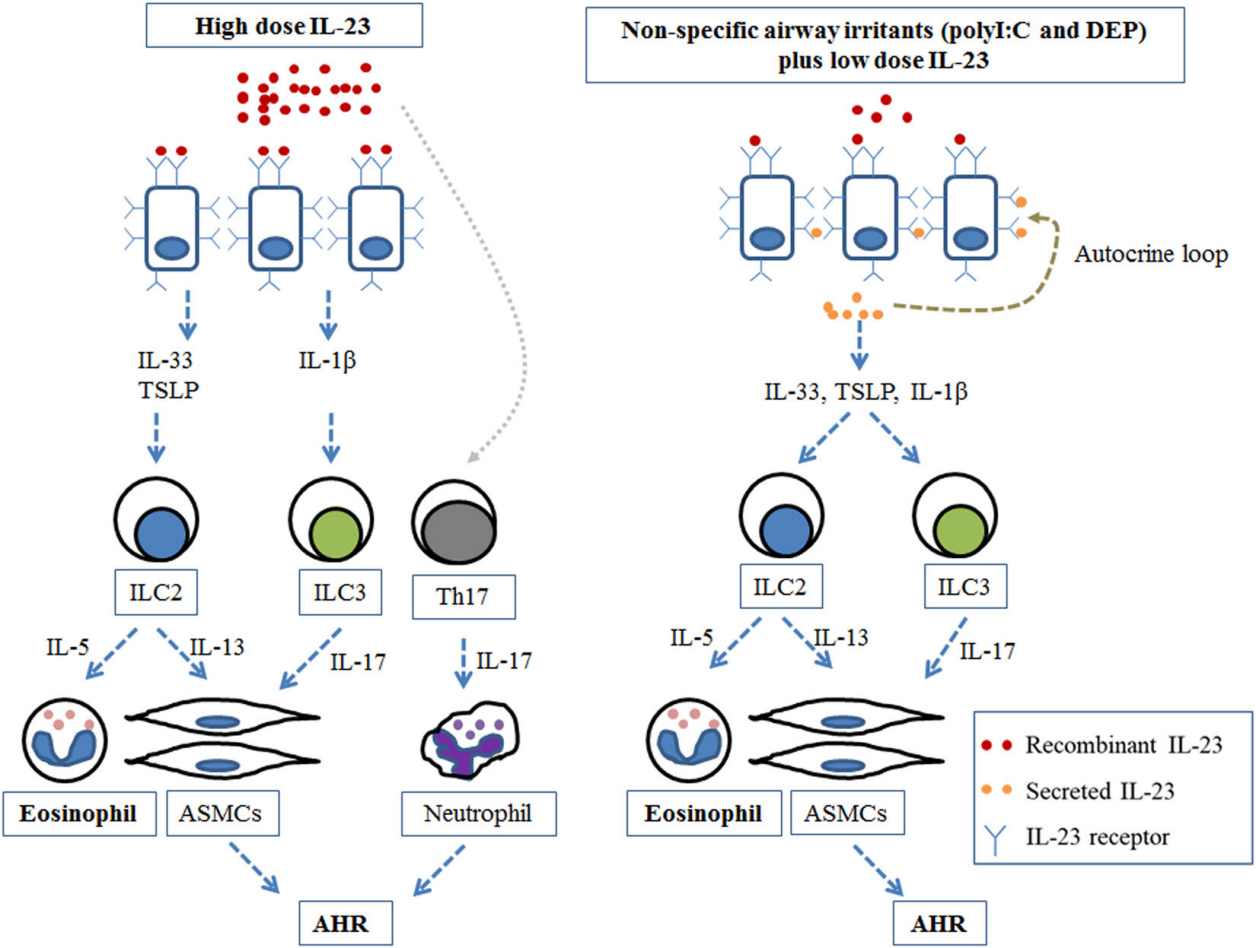
A plausible mechanism for the development of nonallergic eosinophilic asthma. **a** Airway epithelial cells stimulated by high doses of IL-23. High-dose IL-23 binds to IL-23R and induces the secretion of IL-33, TSLP, and IL-1β from airway epithelial cells; these factors can activate ILC2s and ILC3s. Activated ILC2s and ILC3s cause eosinophilic airway inflammation and AHR. In addition, high-dose IL-23 contributes to the development of AHR by directly activating Th17 cells and neutrophils. **b** Airway epithelial cells stimulated by low doses of IL-23 plus nonspecific airway irritants (polyI:C or DEPs). Low-dose IL-23 plus nonspecific airway irritants induce the secretion of a small amount of IL-23 from airway epithelial cells and increases the expression of IL-23R on airway epithelial cells. The secreted IL-23 forms a positive autocrine loop by binding to the increased IL-23R, thereby inducing secretion of IL-33, TSLP, and IL-1β (as with the high-dose of IL-23). Activated ILC2s and ILC3s cause eosinophilic airway inflammation and AHR. ASMC airway smooth muscle cell; AHR airway hyperresponsiveness.

NAEA is thought to be a distinct subtype of asthma. However, there have been few studies on the development of NAEA. Here, we demonstrated that intranasal administration of rIL-23 plus a low dose nonspecific airway irritants (polyI:C or DEPs) without allergens resulted in AHR and eosinophilic inflammation in mice, which are characteristic features of asthma. rIL-23 plus a nonspecific airway irritants induced the release of innate cytokines from airway epithelium (e.g., IL-33, TSLP, and IL-1β), which activated ILC2s and ILC3s. ILC2s and ILC3s, but not CD4+ T cells, were important for the development of NAEA in our experimental models. In addition, increased IL-23R expression in airway epithelial cells was observed, which suggested that a positive autocrine loop might exist in our murine model of NAEA. A plausible mechanism for NAEA based on our observations is presented in Fig. 8.

**Fig. 9:**
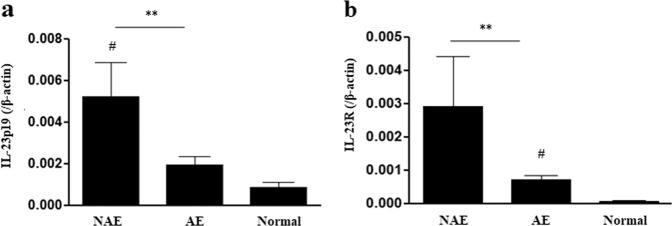
*IL-23A* and *IL-23R* gene expression levels in induced sputum obtained from patients with nonallergic eosinophilic asthma, allergic eosinophilic asthma, and normal controls. **a** IL-23A. **b** IL-23R. ^**^P < 0.01 between two groups; ^#^*P* < 0.05 compared to normal controls; NAEA nonallergic eosinophilic asthma; AEA allergic eosinophilic asthma (details are provided in the supplementary information).

An unexpected but interesting finding of this study was that the administration of 1 μg/mouse rIL-23 for 2 weeks induced characteristics of NAEA in mice without any additional stimuli (Fig. 1). Because IL-23 is essential for the survival and functional maturation of Th17 cells^22^, the role of IL-23 in the pathogenesis of asthma has been intensively evaluated from the perspectives of the IL-23 and Th17 axes, focusing on neutrophilic airway inflammation^23^. However, in an allergic model of murine asthma, IL-23 produced by dendritic cells (DCs) at the site of antigen sensitization facilitated eosinophilia and Th2 immune responses (DC-secreted IL-23 and Th2 cell interaction mechanism);^24^ in addition, increased IL-23 expression in the lungs upon antigen inhalation enhanced the recruitment of both eosinophils and neutrophils^13^. In the present study, the administration of 1 μg/mouse rIL-23 induced the secretion of IL-33 and TSLP from airway epithelium, which subsequently increased the number of ILC2s. In addition, IL-23 was secreted from the airway epithelium. Importantly, IL-5-producing ILC2s are known to mediate eosinophil activation and survival^25^. Thus, our findings suggest that high doses of rIL-23 without allergens may result in eosinophilic airway inflammation due to interactions between epithelium-derived innate cytokines and ILC2s (airway epithelium-secreted IL-23 and ILC interaction mechanism, Fig. 8). Moreover, ILC2s, ILC3s, and Th17 cells, which were induced by the administration of 1 μg/mouse rIL-23, may contribute to the development of AHR (Fig. 8)^26,27^. Given that the innate immune response of airway epithelial cells to nonspecific irritants is important in the development of eosinophilic inflammation in nonallergic asthma^7^, we designed a murine model of asthma based on treatment with low dose polyI:C or DEPs plus a low dose (0.1 μg/mouse) of rIL-23. We consider this model to be highly relevant to the disease that occurs in humans. In accordance with our assumption, the expression levels of *IL23A* and *IL23R* genes were significantly higher in induced sputum from patients with NAEA than in induced sputum from patients with allergic eosinophilic asthma (Fig. 9, details are presented in the supplementary information). This was a cross-sectional measurement, and it is unclear whether these observations were causes or consequences of NAEA in these patients. Previous reports showed that genetic variations in the expression levels of *IL12B* and *IL23R* conferred susceptibility to some immunologic diseases by increasing IL-23 levels in blood or IL-23R expression levels in peripheral blood mononuclear cells^28–30^. We hypothesize that continuous exposure to low doses of nonspecific airway irritants in subjects with enhanced IL-23 signaling, due to genetic variations, may produce the features of NAEA. This genetic susceptibility, combined with different levels of exposure to nonspecific airway irritants, may partly explain why only some individuals in a population develop NAEA, although all members of the population may be continuously exposed to nonspecific airway irritants in daily life. Further studies are needed to confirm this hypothesis.

We recently reported that IL-23 was secreted from airway epithelial cells in a murine model of allergic asthma elicited by house dust mite allergen exposure and administration of IL-33, an epithelium-derived innate cytokine, following activation of IL-23R signaling^31,32^. Notably, polyI:C/rIL-23 or DEP/rIL-23 treatment increased IL-23R expression in mouse lung homogenates and in CD45− EpCAM+ cells gated by flow cytometry in the present study. This observation suggests that a positive autocrine loop may exist. Similar to previous reports^33,34^, the administration of polyI:C or DEPs induced the expression of TSLP or IL-33 from airway epithelial cells in our experiment. Distinct patterns of TSLP or IL-33 secretions might be due to the difference in the amount of IL-23 induced by polyI:C or DEPs or to the intrinsic difference of polyI:C or DEPs as nonspecific irritants, although further studies investigating signal pathways are warranted. We considered this to be partly mediated by IL-23 signaling, because the secretion of TSLP or IL-33 was significantly (but not completely) attenuated by treatment with anti-IL-23p19 antibodies. We further confirmed this by observing that expression levels of TSLP or IL-33, which were induced by administration of polyI:C or DEPs plus rIL-23, showed further increases in IL-23R-overexpressing MLE12 cells (Fig. 7). Thus far, IL-23 has been considered to be primarily secreted by activated DCs or macrophages, whereas IL-23R is reportedly only expressed in T cells and other lymphocytes^35^. IL-23 secretion from DCs or macrophages has an important role in antituberculosis, antiviral, or anti-parasitic immune responses through the induction of Th17 cells^36–38^. However, changes in IL-23 signaling in the airway epithelium that occur during viral infection may play different roles, thus promoting the development of NAEA.

ILC2s, which are activated by TSLP and IL-33, appeared to play important roles in both the polyI:C/rIL-23 and DEP/rIL-23 models, whereas ILC3s, which are activated by IL-β1, were important only in the DEP/rIL-23 model. IL-5-producing ILC2s are known to mediate airway eosinophilia, and IL-13-producing ILC2s are known to induce smooth muscle thickness through IL-13 secretion, thereby increasing AHR^9^, ILC3s are also known to contribute to enhanced AHR^23,27^. When combined with the prior literature, the present findings indicate that polyI:C or DEPs together with IL-23 secreted from airway epithelial cells may induce expression of TSLP, IL-33, and IL-β1, as well as the activation of ILC2s and ILC3s. These factors contribute to the development of NAEA, which might be potentiated by an autocrine loop. One limitation to note is that we did not perform experiments using mice from which ILC2s were completely removed. To confirm our findings, a further study with ILC2 knock-out mice will be essential. In addition, the effect of disrupting the IL-23/IL-23R pathway in the murine models in this study need to be evaluated. We focused on the role of the IL-23/IL-23R pathway in the airway epithelium, but it is not easy to develop a model with airway epithelium-specific disruption of the IL-23/IL-23R pathway. This would be another limitation in generalizing our observations.

Neutrophil increases in BAL fluid of the polyI:C/rIL-23 and DEP/rIL-23 models (Figs. 2 and 3) are worthy of being discussed. Exposure to polyI:C and DEPs, nonspecific airway irritants, may result in inflammation in the airway. Accordingly, previous reports showed that the administration of polyI:C or DEPs alone to the airways of mice, even at a low dose, increased neutrophils in the airways^20,39^. However, eosinophils did not increase in the airways of these murine models. A noble finding of the present study was that airway eosinophilia developed when rIL-23 was added to polyI:C or DEP, which suggests rIL-23 has its own role in the pathogenesis of NAEA. The number of neutrophils in BAL fluid did not differ significantly between the polyI:C and polyI:C/rIL-23 groups, or between the DEP and DEP/rIL-23 groups. In addition, CD4+IL17+ cells did not increase significantly in the polyI:C/rIL-23 and DEP/rIL-23 groups, which differed from the effects in mice treated by a high dose of rIL-23 only. In addition, the number of neutrophils in the BAL fluid of our model was 4–10 × 10^3^/mL, whereas 7–12 × 10^4^/mL were detected in previous murine models of the neutrophilic asthma related to the IL-17 pathway^40,41^. Based on these findings, we believe that increased neutrophils observed in this study were an epiphenomenon related to nonspecific airway irritations.

In conclusion, we found that mice that had undergone intranasal administration of rIL-23 plus a low dose of nonspecific airway irritants (polyI:C or DEPs) without allergens exhibited AHR and eosinophilic airway inflammation similar to NAEA in humans. Of course, other nonspecific airway irritants, such as bacteria-derived extracellular vesicles, may play a role in the development of NAEA in subjects with susceptibility. Airway microbiota composition has been shown to regulate immune responses, and this link may be partly mediated by extracellular vesicles from microbiota^42^. All these possibilities need to be tested by additional research in the future.

## Supplementary information

Supplementary Information
