# Supplementary material for: Role of interleukin-23 in the development of nonallergic eosinophilic inflammation in a murine model of asthma
Source: Exp Mol Med. 2020 Jan 20;52(1):92–104. doi: 10.1038/s12276-019-0361-9 (PMC7000690; doi:10.1038/s12276-019-0361-9)

**Supplementary Information**

**Role of interleukin-23 in the development of nonallergic eosinophilic inflammation in a murine model of asthma**

Hyun Seung Lee^1^, Da-Eun Park^1^, Ji-Won Lee^2^, Kyung Hee Sohn^3^, Sang-Heon Cho^1,4^, Heung-Woo Park^1,4^

^1^Institute of Allergy and Clinical Immunology, Seoul National University Medical Research Center, Seoul, Republic of Korea

^2^Division of Allergy and Clinical Immunology, Department of Asan Medical Center, University of Ulsan College of Medicine, Seoul, Republic of Korea

^3^Department of Internal Medicine, Kyung Hee University Medical Center, Seoul, Republic of Korea

^4^Department of Internal medicine, Seoul National University College of Medicine, Seoul, Republic of Korea

**METHODS**

**Airway hyperresponsiveness measurement**

Measurement of dynamic resistance was performed using a Flexivent system (Scireq, Montreal, Canada). Mice were anesthesia with ketamine (90 mg/kg body weight) and xylazine (10 mg/kg body weight), tracheostomized and connected to the flexivent ventilator via a 19-gauge cannula. Mice were ventilated using following settings; tidal volume of 10 ml/kg body weight, 150 breaths/minute, positive end-expiratory pressure 3 cm H2O. Mice were allowed to stabilize for 5 min before measurements started. Measurement of airway resistance (cm H2O/ml/s) was determined using snapshot-150 perturbation. MCh (acetyl-b-methylcholine chloride; Sigma-Aldrich) provocation testing started with PBS, followed by MCh aerosols with increasing concentrations (0, 12.5, 25, 50, and 100 mg/ml). The graphs show values of 50 mg/ml and 100 mg/ml.

**ILC2 cell sorting**

Mice were treated with recombinant IL-33 (0.5 μg/mouse; eBioscience) for 5 days. Lungs were harvested for ILC2 cell sorting; lineage-negative cells were gated as cells that did not express FITC-conjugated CD3, CD4, CD8, CD11b, CD11c, CD19, F4/80, FcεRI, and CD49b. PE-conjugated ST2 and APC-conjugated ICOS were also used for sorting. The lungs of several mice were pooled for ILC2 cell sorting. A FACSAria II was used (BD Biosciences) for cell sorting. Sorted lung ILC2s (1 × 10^4^/well) were expanded in vitro using 10% FBS RPMI complete medium containing IL-2 (10 ng/mL; BD Biosciences) and IL-33 (10 ng/mL, R&D) for 7 days.

***IL23A* and *IL23R* gene expression in induced sputum obtained from patients with asthma**

A total of 17 patients with NAEA (male:female = 3:14, mean age [standard deviation] = 54.3 [15.7]), 63 patients with AEA (male:female = 11:52, mean age [standard deviation] = 55.2 [10.4]), and 13 normal controls (male:female = 4:9, mean age [standard deviation] = 47.4 [17.3]) were enrolled at Seoul National University Hospital. A diagnosis of asthma was made when a subject with symptoms of dyspnea or wheezing showed reversible airway obstruction after bronchodilator inhalation (200 mL and 12% or more improvement in forced expiratory volume in 1 second) or positive methacholine AHR. Patients with asthma who showed positive skin prick test results or *in vitro* specific-IgE test results when exposed to a panel of common allergens were regarded as “allergic”; otherwise, they were regarded as “nonallergic.” A normal control was defined as a subject who had no respiratory symptoms and showed negative results in both methacholine AHR test and skin prick test or *in vitro* specific-IgE test when exposed to a panel of common allergens. Processing of induced sputum was performed as previously described (*Jung JW, et al. Tohoku J Exp Med 2014;233:49-56*). Sputum eosinophilia was defined as the proportion of eosinophils greater than 3% of the total inflammatory cells. The cell pellet was mixed with Trizol (Gibco, Grand Island, NY, USA) for RNA extraction. After, RNA extraction, real-time polymerase chain reaction (RT-PCR) was performed using an RT-PCR kit (Promega, Madison, WI, USA). The mRNA expression levels of IL-23A (IL-23p19) and IL-23R were amplified using an ABI 7500 real-time PCR system and SYBR Green master mix (Applied Biosystems, Foster City, CA, USA). Expression levels of each gene within each sample were normalized against β-actin using the formula 2^−ΔΔCt^, in which ΔΔCt = (Ct mRNA – Ct β-actin) and Ct is the cycle threshold. The primer sequences for IL-23A and IL-23R were as follows:

IL-23A Fwd: CTC AGG GAC AAC AGT CAG TTC

IL-23A Rev: ACA GGG CTA TCA GGG AGC A

IL-23R Fwd: AGC AGC AAT TAA GAA CTG CCA

IL-23R Rev: TAC CAA AGC CGA GCT GTT GTT

**SUPPLEMENTARY FIGURE LEGENDS**

**Figure S1. Experimental protocol**

polyI:C or DEP plus 0.1 μg/mouse rIL-23 were intranasally administered to mice (on days 1, 2, 3, 14, 15, 21, and 22) and methacholine airway hyperresponsiveness was measured at 24 h after the last instillation. MBPT, methacholine bronchial provocation test.

**Figure S2. Gating strategy for flow cytometry analysis**

Cells were first gated for lymphocytes (SSC-A vs. FSC-A) and singlets (FSC-H vs. FSC-A). a Singlets were further analyzed for CD4− versus CD4+ cells. b IL-13 or IL-17 intracellular expression was then determined in CD4+ cells. c For gating of ILC3s, IL-17 expression was determined in CD25+Lin-CD4−cells. d, e For gating of ILC2s, IL-13 or IL-5 expression was determined in ICOS+ Lin−cells. f For gating of eosinophils from BAL fluid, Siglec F expression was determined in CD11b+ CD11c−cells. IL-23R expression was determined in CD45−and CD45+ cells. g IL-23R expression was determined in EpCAM+ CD45−cells. H–I. IL-23R expression was determined in F4/80− CD45+or CD4+ CD45+ cells.

**Figure S3. ILC2 sorting**

a Experimental protocol (0.5 μg/mouse rIL-33 was intranasally administered to mice for 5 consecutive days). b To isolate ILC2s, ICOS+ ST2+ Lin− cells were identified.

**Figure S4. Detection of IL-23R overexpression in MLE12 cells**

Primary antibodies used were IL-23R (1:1000; R&D Systems) and β-actin (Abcam).

**Figure S5. FACS plots of mice treated by rIL-23**

a, b Gating of ILC2s, IL-13 or IL-5 expression was determined in ICOS+ in Lin- CD4−cells. c Gating of ILC3s, IL-17 expression was determined in CD25+ in Lin-CD4−cells. d, e IL-13 or IL-17 intracellular expression was determined in CD4+ cells.

**Figure S6. FACS plots of polyI:C/rIL-23 model**

a Gating of eosinophils from BAL fluid, Siglec F expression was determined in CD11b+ CD11c−cells. b, c Gating of ILC2s, IL-13 or IL-5 expression was determined in ICOS+ in Lin- CD4−cells. d Gating of ILC3s, IL-17 expression was determined in CD25+ in Lin-CD4−cells. e IL-13 or IL-17 intracellular expression was determined in CD4+ cells.

**Figure S7. FACS plots of DEP/rIL-23 model**

a Gating of eosinophils from BAL fluid, Siglec F expression was determined in CD11b+ CD11c−cells. b, c Gating of ILC2s, IL-13 or IL-5 expression was determined in ICOS+ in Lin- CD4−cells. d Gating of ILC3s, IL-17 expression was determined in CD25+ in Lin-CD4−cells. e IL-13 or IL-17 intracellular expression was determined in CD4+ cells.

**Figure S8. IL-23R expression in cells from mouse lung tissue**

a IL-23R+ EpCAM+ CD45− cells. b CD11+ F4/80− CD45+ cells. c CD4+ CD45+ cells. ^*^*P* < 0.05 between groups; ^#^*P* < 0.05 compared to the control mice.

**Figure S1.**


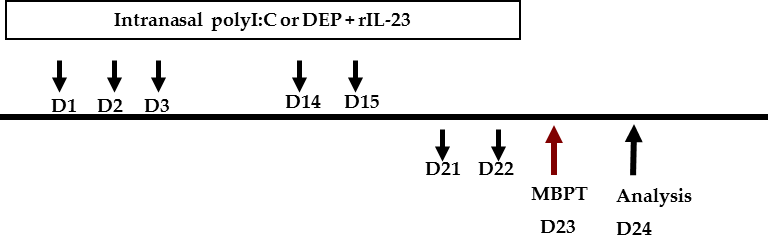


**Figure S2.**

**
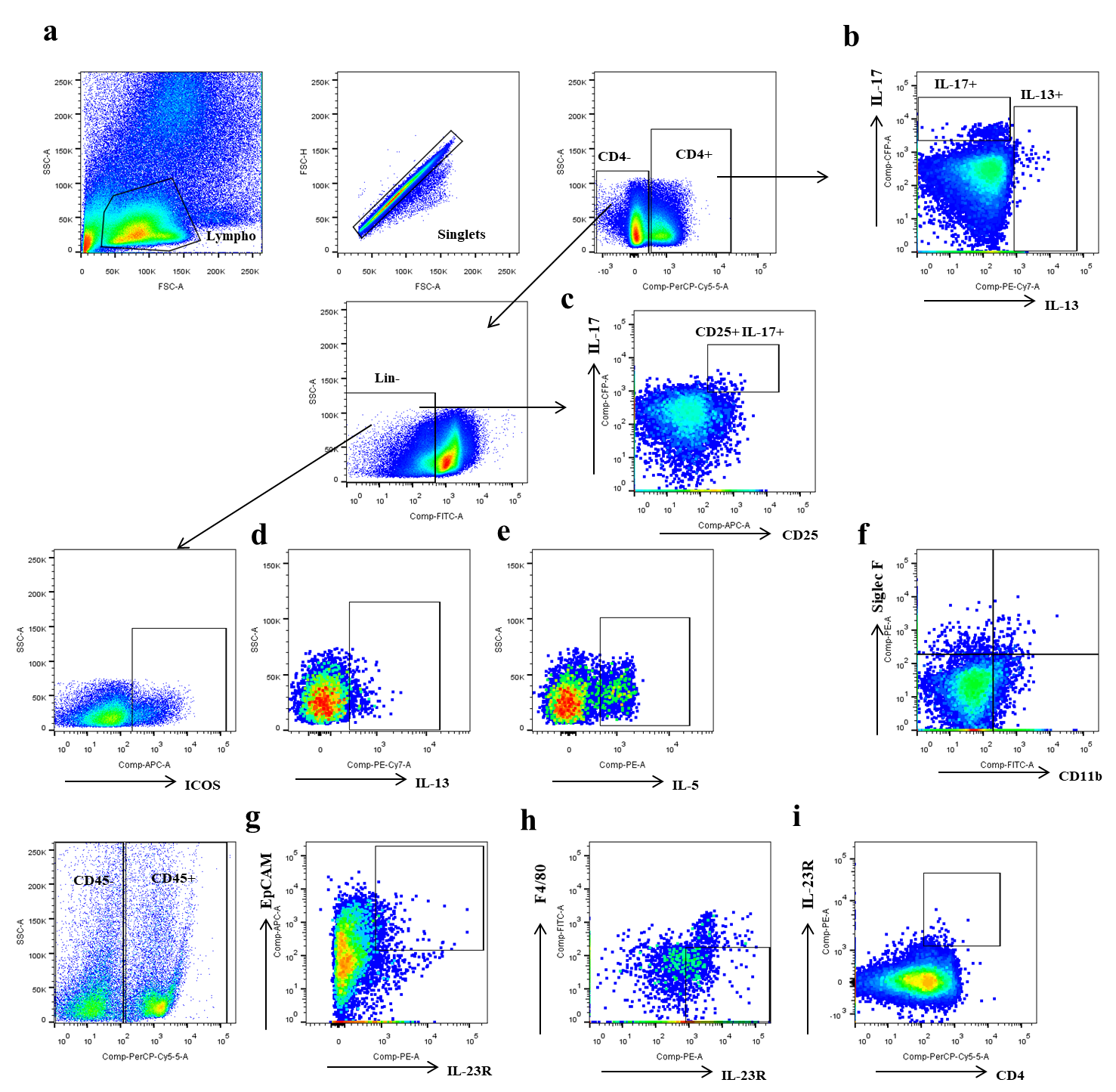
**

**Figure S3.**


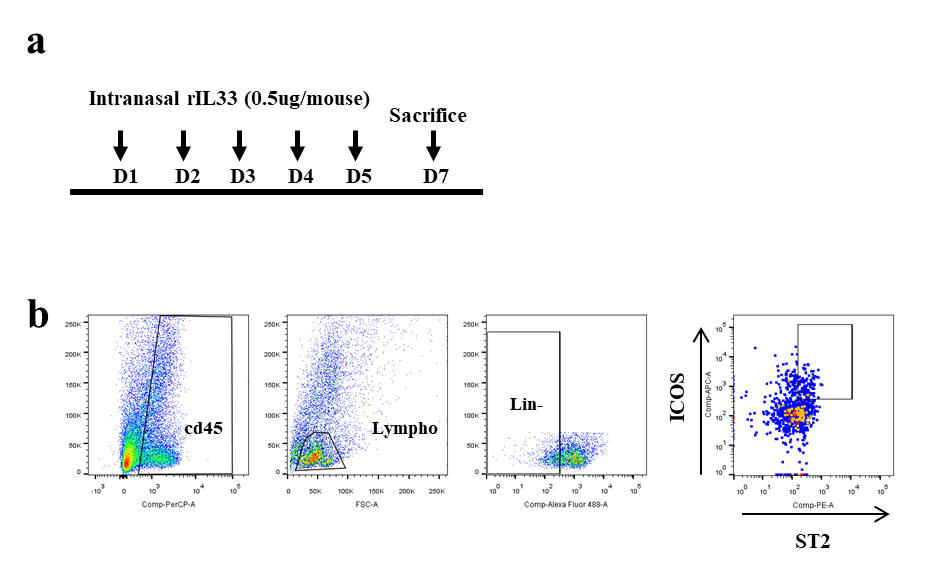


**Figure S4.**


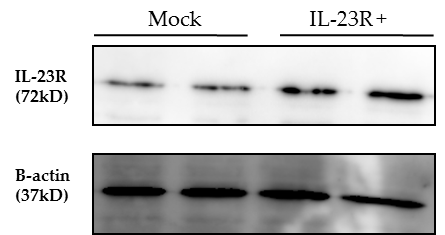


**Figure S5.**

**
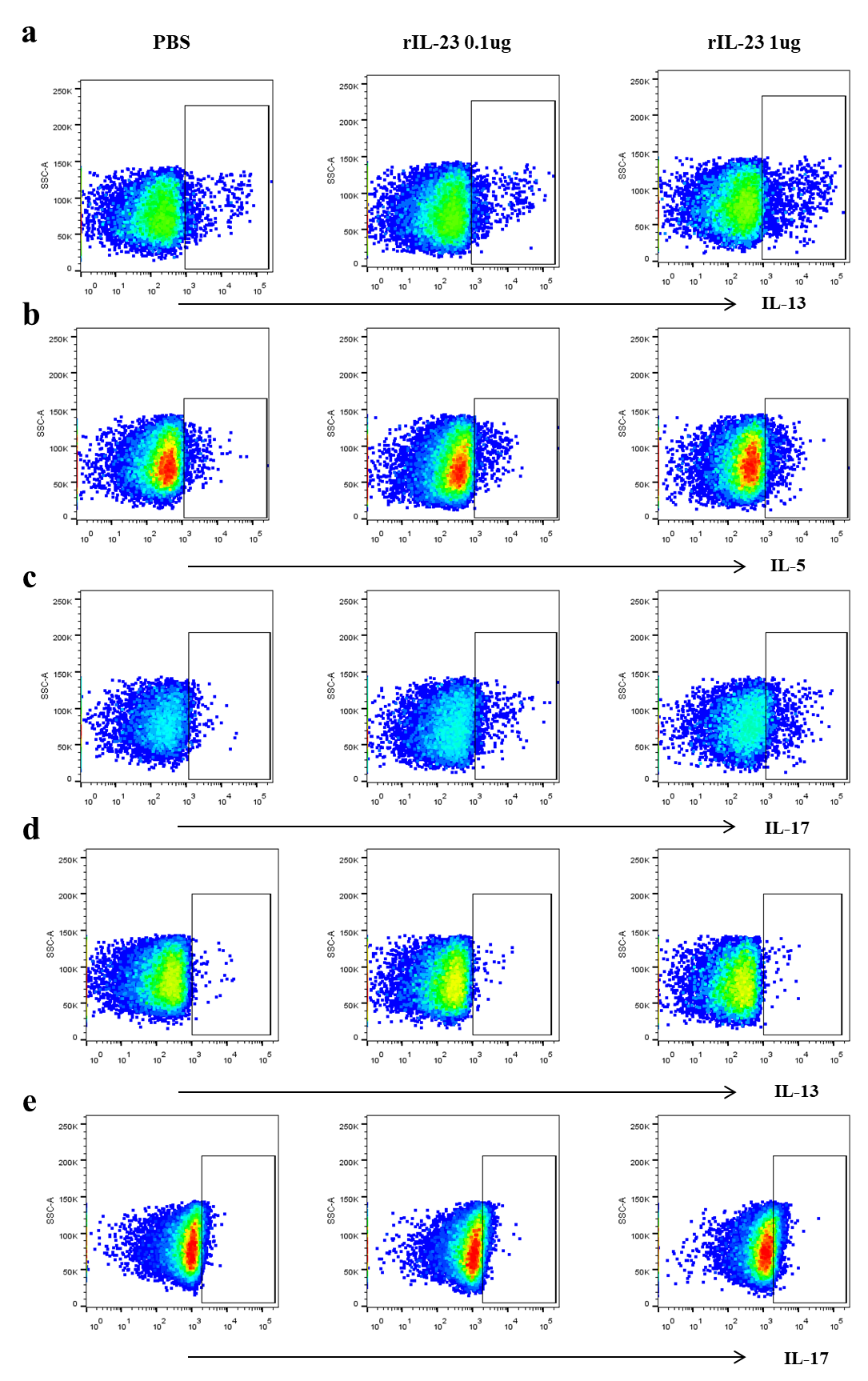
**

**Figure S6.**

**
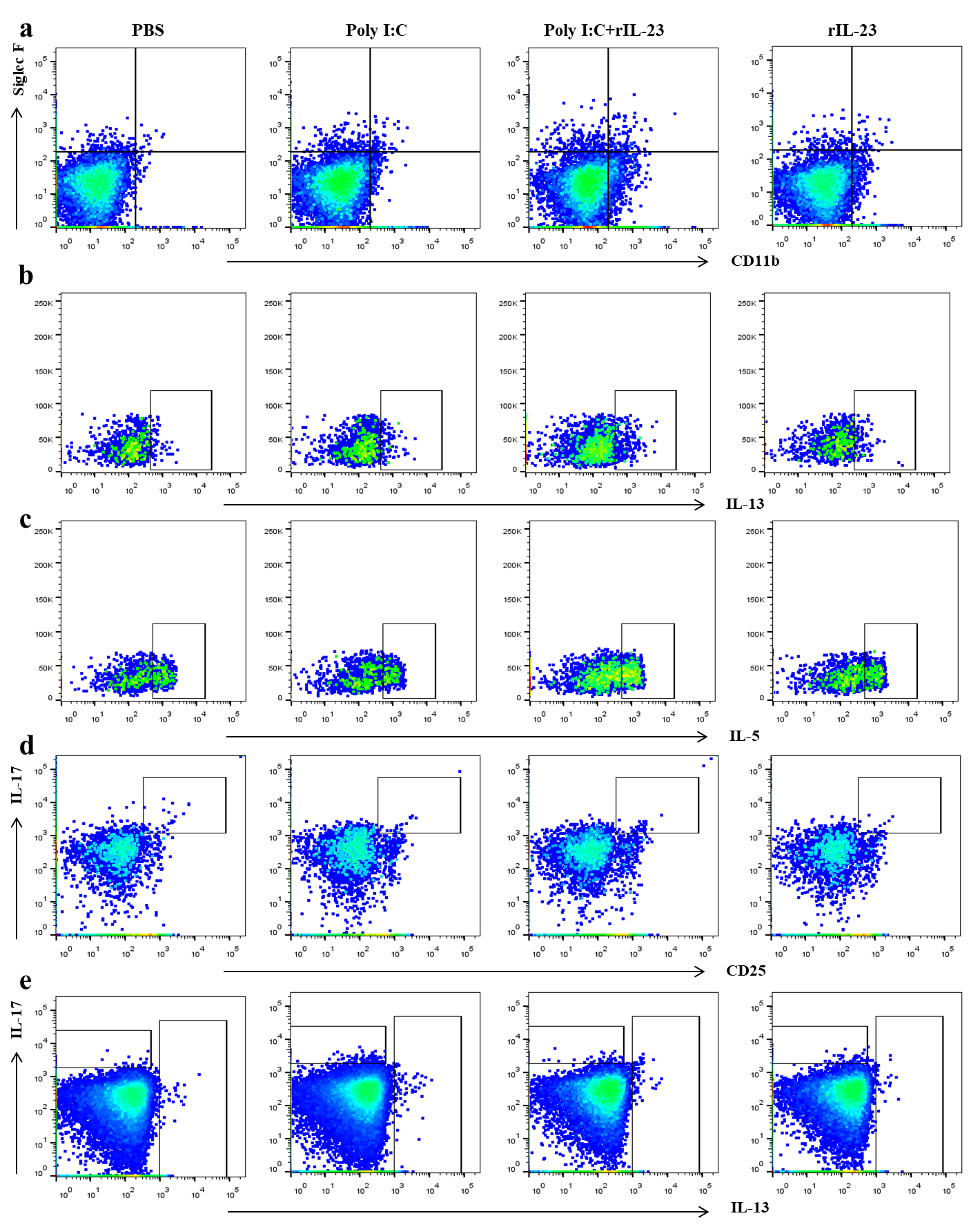
**

**Figure S7.**

**
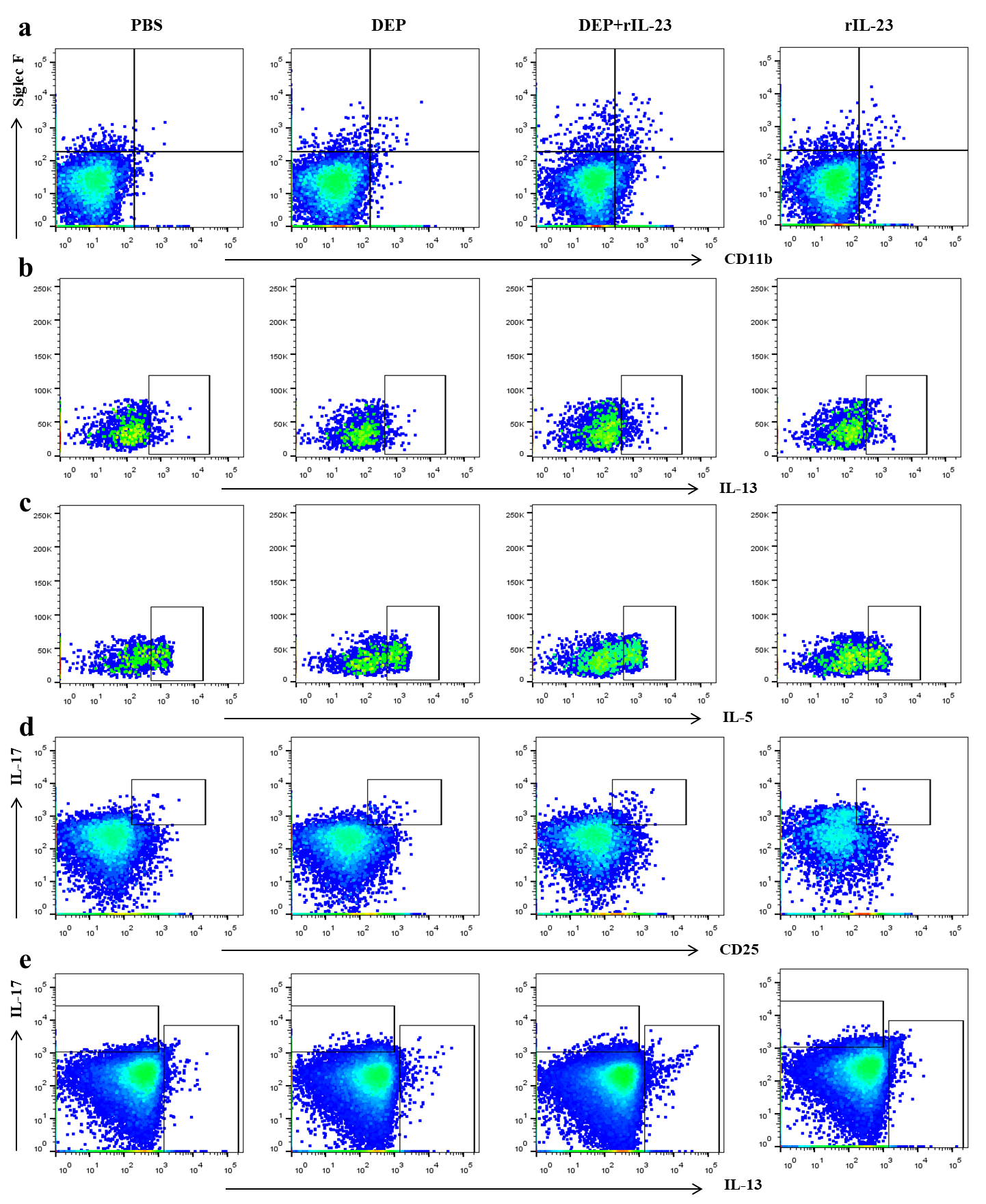
**

**Figure S8.**


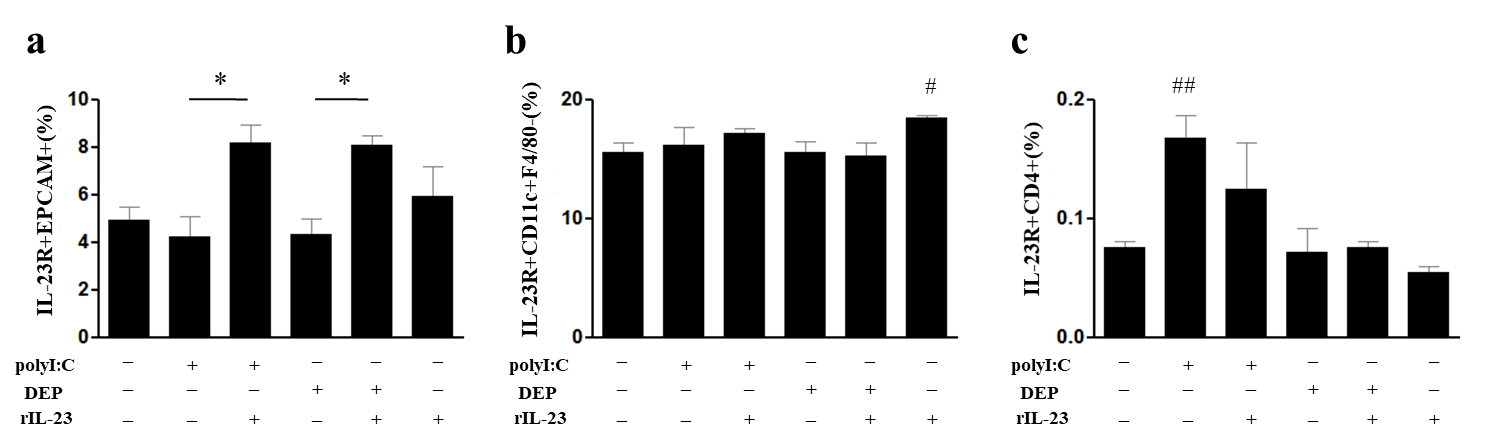

Supplement: Supplementary file 1 — Supplementary Information [file 12276_2019_361_MOESM1_ESM.docx]
